# GDNF enhances HGF-induced tubulogenesis and organization of Sertoli cell

**DOI:** 10.1007/s10815-025-03493-7

**Published:** 2025-05-22

**Authors:** Begum Durkut-Kuzu, Ciler Celik-Ozenci

**Affiliations:** 1 Graduate School of Health Sciences, Reproductive Medicine, Koc University, Istanbul, Turkey; 2 Department of Histology and Embryology, School of Medicine, Koc University, Istanbul, Turkey; 3 Research Center for Translational Medicine (KUTTAM), Koc University, Istanbul, Turkey

**Keywords:** Sertoli cell, Matrigel-based 3D culture, GDNF, HGF, In vitro tubule-formation

## Abstract

**Purpose:**

Hepatocyte growth factor (HGF), secreted by Sertoli cells, activates the c-Met receptor, facilitating seminiferous tubule formation. Glial cell-derived neurotrophic factor (GDNF) promotes embryonic Sertoli cell proliferation and cord formation. This study aimed to investigate whether exogenous GDNF contributes to HGF-induced in vitro organization of Sertoli cells in a Matrigel-based three-dimensional (3D) culture system.

**Methods:**

Four experimental groups were established using the 15P-1 Sertoli cell line: control, HGF-treated, GDNF-treated, and combined HGF- and GDNF-treated. Tubular structure length and branching were quantified using image analysis software, while the expression of ZO-1 protein was assessed via immunofluorescence staining in 3D Matrigel-based culture for 5 days. Additionally, *Gfrα-1*, *Ret*, *Ncam*, and *Met* mRNA expression in Sertoli cells were analyzed. The expression levels of ZO-1, c-MET, and p-c-MET were analyzed in two-dimensional (2D) culture after 5 days.

**Results:**

Quantitative analysis revealed a significant increase in the length and branching of tubular-like structures when GDNF was co-administered with HGF, compared to other groups. Additionally, there was a notable increase in ZO-1 protein expression with the combined administration of GDNF and HGF, surpassing levels observed in the control group. Furthermore, co-administration of HGF and GDNF significantly elevated phospho-c-Met levels compared to the control group.

**Conclusion:**

Our study reveals that exogenous GDNF enhances the HGF-induced tubular organization of Sertoli cells in vitro. The concurrent administration of GDNF and HGF markedly augments the formation of tubular structures and the expression of the ZO-1 protein, indicating a potential synergistic influence on the organizational and signaling cascades within Sertoli cells.

**Supplementary Information:**

The online version contains supplementary material available at 10.1007/s10815-025-03493-7.

## Introduction

Understanding the testicular organization process is essential in conserving male fertility and contributing to treatment processes. Recently, research using cell-tissue culture, biomaterials, and bioactive factors has revealed the cells, factors that play a role in this process, and their relationships [1]. However, there is not yet a technique that can fulfill the needs of patients with azoospermia who do not have spermatids in their testes or prepubertal patients whose germ cells are depleted after chemotherapy. Therefore, in vitro studies are in demand, especially for a better understanding of testicular organization.

Seminiferous tubule formation is a highly coordinated process that begins during embryonic development and extends into the postnatal period [2]. In both humans and mice, the transition of gonocytes into spermatogonia, their migration, and subsequent arrangement along the basal membrane of Sertoli cells commence prenatally and finalize postnatally [3, 4]. During embryogenesis, Sertoli cells aggregate to form seminiferous cords, encapsulating gonocytes that will later differentiate into spermatogonia [3]. As development continues postnatally, Sertoli cells proliferate, supporting the maturation of seminiferous tubules, while gonocytes re-enter the cell cycle and migrate toward the basal membrane, linking prenatal and postnatal development [4]. This tightly regulated process is crucial for male fertility, and any disruptions can result in impaired spermatogenesis. Vascular endothelial cells in the testis guide the formation of seminiferous cords by aligning parallel to migrating vascular endothelial cells from the mesonephros during the postnatal period, playing a crucial role [5]. Fibroblast growth factor (FGF) signaling and HGF directly or indirectly influence the formation of seminiferous tubules [6, 7]. Defects in testicular organization have been linked to conditions such as non-obstructive azoospermia and Sertoli cell-only syndrome, leading to infertility [8]. Understanding these mechanisms could aid in developing regenerative therapies for male reproductive disorders.

On the other hand, in vitro testicular organization consists of two main parts, in which several cell types can play a role: testicular cord-like structures and tubule-like structures [9]. The organization proceeds in the same way in both 2D and 3D culture models with Sertoli cells which play a major role during in vivo as well [9]*.* Firstly, the most important step is to provide “confluence” with the interaction of cells with each other. In the second stage, Sertoli cells are collected in multi-nodular clusters. Cord-like structures are formed by the connections formed by the clusters of cells with each other. Tubule-like structures complete the final stage with lumen formation by establishing the polarity of Sertoli cells. To understand testicular development and physiology, 2D and 3D culture models have been utilized with Sertoli cells and other testicular cells and by cell–cell and cell-ECM interaction between them [10].

Advanced cell organization and cell viability are observed in a Matrigel-based culture system [9]. While the efficiency of spermatogenesis in 3D culture systems has been limited by factors such as the absence of full testicular-specific architecture [11], Matrigel’s properties make it a valuable tool in examining the effects of growth factors on the organization of Sertoli cells and their potential for enhancing in vitro testicular modeling. Due to its ability to regulate cellular polarity formation, Matrigel™ is suitable for studies focused on the formation of Sertoli cell-derived tubule-like structures [11].

HGF plays a role as an important part of the paracrine signaling pathway in the process of testicular tubulogenesis [12]. HGF produced by mesenchymal and somatic cells promotes to the formation of seminiferous tubules by Sertoli cells expressing the c-Met receptor and regulates tubule localization of Sertoli cells [13]. In 1999, a study demonstrated that HGF is required in the process of epithelial tubulogenesis in vitro using the Sertoli cell line and primary Sertoli cells [14].

The glial cell-derived neurotrophic factor is an important paracrine factor that is secreted by Sertoli cells, peritubular myoid (PTM) cell, and testicular vascular cells and binds to the GFRα1/Ret receptor complex and is a member of the transforming growth factor-beta (TGF-ß) superfamily [15]. GDNF has been identified as critical to maintaining the SSCs [15]. In a 2003 study, it was shown that GDNF induced ureteric branching of the Madin-Darby canine kidney (MDCK) cells in culture by activating the HGF receptor Met, without using Ret signaling [16]. Remarkably, GDNF was demonstrated in 2019 that HGF increases the capacity of renal tubule forming in vitro [17].

Three-dimensional (3D) testicular culture models have greatly advanced our understanding of seminiferous tubule formation by better mimicking the testicular microenvironment and the regulatory mechanisms of spermatogenesis. While GDNF is well known for its role in spermatogonial stem cell (SSC) self-renewal and maintenance, its potential influence on Sertoli cell-mediated testicular organization remains unexplored. In this study, we demonstrate that exogenous GDNF enhances hepatocyte growth factor (HGF)-induced in vitro organization of Sertoli cells within a Matrigel-based 3D culture system, providing new insights into testicular organization and potential therapeutic implications for male infertility.

## Materials and methods

**Fig. 1:**
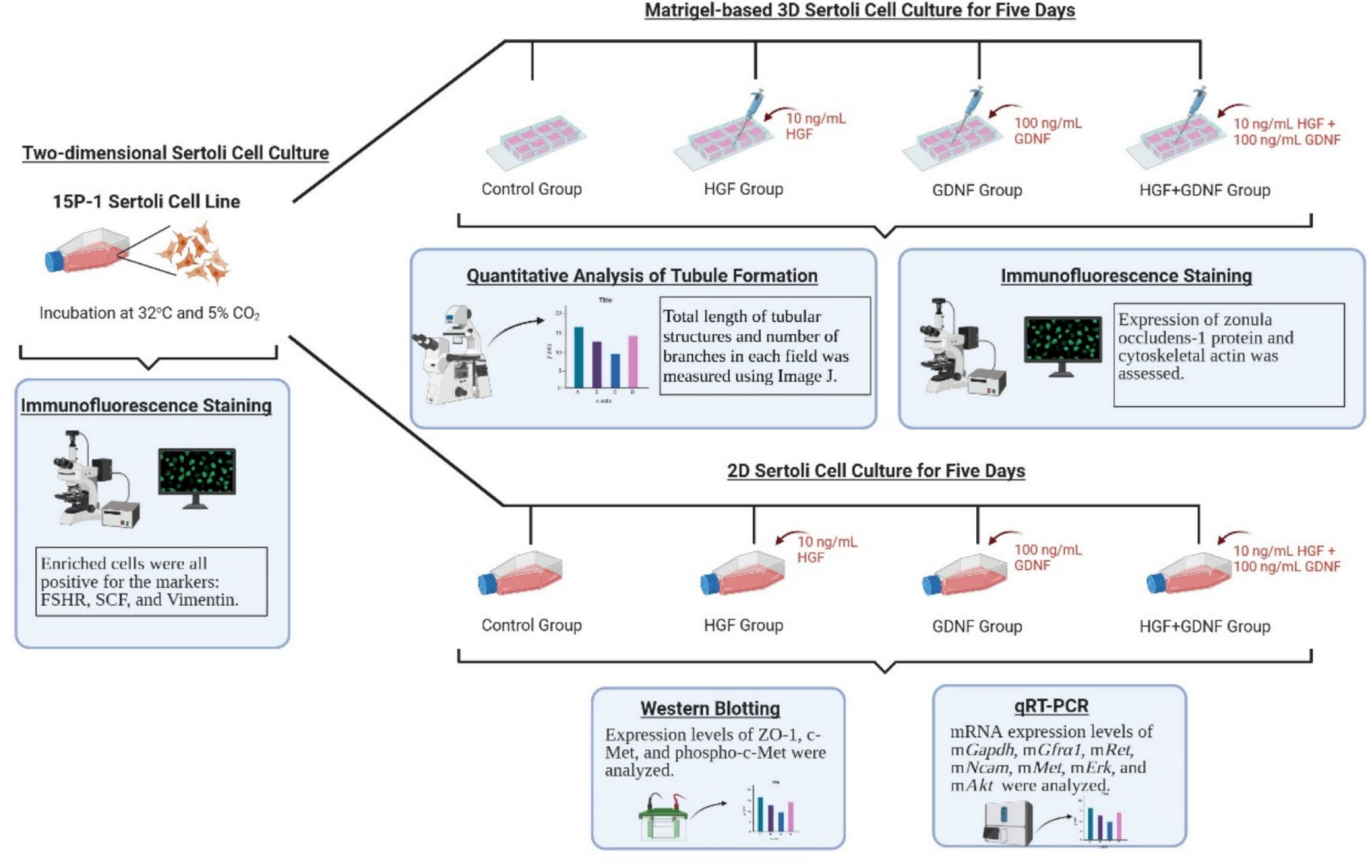
Experimental design. Sertoli cells (15P-1, ATCC CRL-2618) were cultured in DMEM-high glucose with 10% FBS and 1% PS at 32 °C with 5% CO_2_ until 80–90% confluency. Passage fifteen cells were used for all experiments, each performed once and repeated two additional times independently (*n* = 3). Cells were validated for Sertoli cell markers FSHR, SCF, and vimentin via immunofluorescence staining. For 3D culture, cells were seeded on GFR-Matrigel-coated 8-well chamber slides, treated with 10 ng/mL HGF, 100 ng/mL GDNF, or both, and incubated for 5 days. Tubular structures were quantified using ImageJ. ZO-1 expression was analyzed through immunofluorescence staining, and protein levels of c-MET, p–c-MET, and ZO-1 were confirmed by western blotting. Data are presented as mean values ± SEM from three independent experiments, each with triplicate iterations. The illustration was generated using the BioRender software

To investigate the effects of HGF and GDNF on Sertoli cell morphology and intercellular dynamics, we employed the 15P-1 Sertoli cell line. Cells were cultured under standard conditions and used at passage fifteen for all experiments. Validation of Sertoli cell markers was performed via immunofluorescence. For 3D culture, cells were seeded on GFR-Matrigel-coated chamber slides (154,453, Thermo Scientific™) and treated with HGF, GDNF, or their combination, followed by quantitative analysis of tubular structures and assessment of protein expression through immunofluorescence and western blotting (Fig. 1).

### Cell culture

**Fig. 2:**
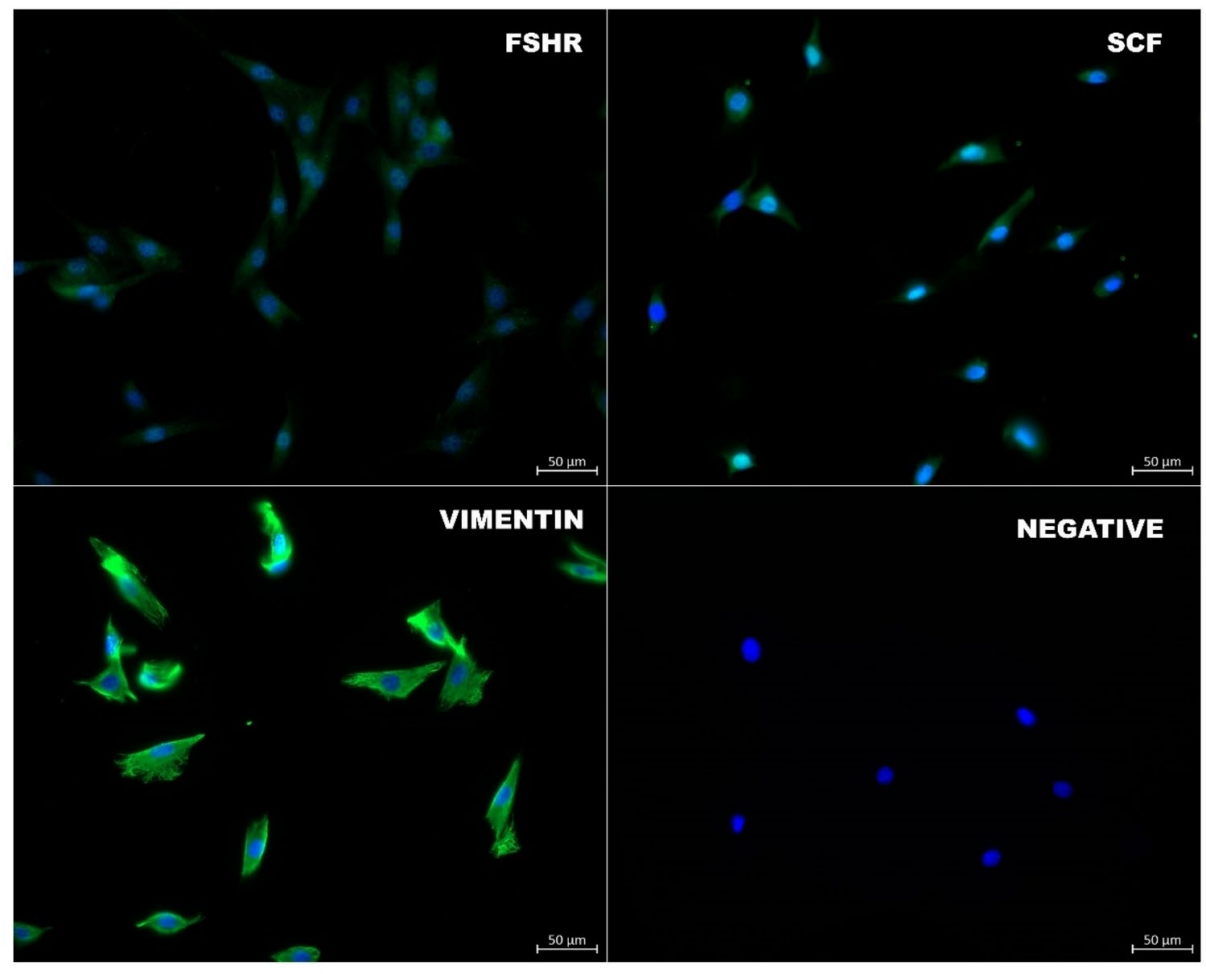
Expression of follicle-stimulating hormone receptor (Fshr), stem cell factor (Scf), and vimentin in Sertoli cells after 72 h at 2D culture

15P-1 Sertoli cell line was purchased from ATCC (CRL-2618) and cultivated as instructed by the supplier. The cells were cultured in Dulbecco’s Modified Eagle’s Medium (DMEM)-high glucose (319–005-CL, Wisent Inc.) supplemented with 10% fetal bovine serum (FBS) (S181H-500, Biowest) and 1% penicillin–streptomycin (PS) (L0022-100, Biowest) at 32 °C in a humidified incubator, under 5% CO_2_. When the cells reached 80–90% confluency, they were passaged according to the guidelines provided by the American Type Culture Collection [18]. All experiments were conducted using cells from passage fifteen, and each experiment was performed once and independently repeated two additional times (*n* = 3). Immunofluorescence staining was carried out using specific antibodies to verify the protein expression of the Sertoli cell marker in the cells. The enriched cells were all positive for the markers using antibodies against follicle-stimulating hormone receptor** (**FSHR) (PA5-99,424, Invitrogen), stem cell factor** (**SCF) (bs-0545R, Bioss In.), and vimentin (ab92547, Abcam), as previously described (Fig. 2).

### Three-dimensional cell culture

The bottoms of the 8-well chamber slide were coated with GFR-Matrigel (5 mg/mL) (354,230, Corning®) and incubated for 30 min at 32 °C and 5% CO_2_ to allow solidification of the gel. Complete cell culture medium was mixed with GFR-Matrigel at a ratio of 2%. The cell suspension was prepared at a density of 3 × 10^5^ cells/cm^2^ in a mixed medium. The culture was incubated for 24 h at 32 °C and 5% CO_2_. Subsequent to a 24-h period, the medium was exchanged with a fresh medium commensurate with the delineated wells assigned to the respective experimental cohorts: 10 ng/mL HGF (HGF Mouse SRP3300-20UG, Sigma-Aldrich) was added for HGF groups, 100 ng/mL GDNF (GDNF Mouse SRP3200-10UG, Sigma-Aldrich) was added for GDNF groups, 10 ng/mL HGF and 100 ng/mL GDNF were added together to form HGF + GDNF groups [14, 17]. All groups were incubated at 32 °C and 5% CO_2_ incubator for 5 days. The cell culture was observed by inverted microscope having phase-contrast every 24 h, including the day of culture (D0).

### Quantitative analysis of tubule formation

Sertoli cells were cultured within gels supplemented with the specified factors. At predetermined time points, quadruplicate high-power fields were randomly selected and captured. The total length of the tubular structures (measured in micrometers) and the frequency of branching events within each field were quantified using ImageJ software (National Institutes of Health, Bethesda, MD). The data presented are the mean values with standard error of the mean (SEM), derived from three independent experimental runs, with four images analyzed for each experimental condition.

### Immunofluorescence staining

Each well was washed with PBS and fixed in 4% paraformaldehyde for 20 min at 37 °C. After fixation, they were permeabilized with 0.5% Triton X-100 in PBS at RT for 10 min. Following the permeabilization step, the wells were rinsed once for 10 min with wash buffer (1X: 130 mM NaCl, 13 mM Na2HPO4, 3.5 mM NaH2PO4, 7.7 mM NaN3, 0.1% bovine serum albumin, 0.2% Triton X-100, 0.05% Tween-20 at pH 7.4.) at RT. After the previous step, they were incubated with 200 µL of 10% goat serum in wash buffer (blocking solution) for 1 h at RT. Next, the primary antibody specifically ZO-1 (bs-1929R, Bioss Inc.; 1:100) diluted in blocking solution were incubated for 2 h at RT. The wells were rinsed three times, 10 min each with wash buffer at RT. Alexa Fluor 488-conjugated anti-rabbit secondary antibody (A-11008, Invitrogen, 1:500), specific for polyclonal rabbit antibodies, was diluted in blocking solution and incubated for 60 min at RT. Additionally, all groups in other wells were incubated with FITC-conjugated phalloidin antibody (P5282, Sigma-Aldrich) for 90 min at 37 °C for visualization of the cytoskeletons. The wells were rinsed three times, 10 min each with wash buffer at RT. Following the final incubation, VECTASHIELD® Mounting Medium with DAPI (H-1200, Vector Laboratories, Inc.) was performed on a slide and examined under a confocal microscope (Leica DMI8 SP8).

### Western blotting

On day 5 of culture, 0.25% Trypsin–EDTA (325–043-EL, Wisent Inc.) was applied to cells, and the cell suspension was obtained from the culture. For the washing step, the supernatant was removed, and the pellet was suspended with cold PBS. The suspension was centrifuged at 1500 rpm and 4 °C for 5 min. The supernatant was removed again, and protein inhibitor cocktail (PIK) (539,136, Sigma-Aldrich) was added to the pellet together with RIPA buffer (89,900, Thermo Scientific™) and incubated on ice for 30 min. After incubation, it was centrifuged at 4 °C and maximum rpm for 10 min. The supernatant containing the proteins was obtained, and amounts of protein were determined by using the BCA (Bicinchoninic Acid) Protein Assay Kit. Then, 3.5 µL marker and 20 µL samples containing 20 µg of protein for each group were loaded on SDS gels and run. Proteins were transferred to a PVDF membrane using the blotting kit (Bio-Rad), blotting solution (Bio-Rad), and the Trans-Blot® Turbo™ Transfer System (Bio-Rad) for 15 min to achieve rapid blotting. Membranes were washed with TBS-T for 10 min and blocked with 5% milk powder at room temperature for 1 h. After blocking, c-met (1:500, bs-0668R, Bioss), p–c-met (1:500, bs-3271R, Bioss), and ZO-1 (bs-1929R, Bioss Inc.; 1:100) were applied and incubated overnight at 4 °C. After incubation, it was washed 3 times with TBS-T for 10 min and incubated with secondary antibody diluted 1:3000 for 1 h at room temperature and then washed. Finally, results were observed using Pierce™ ECL Western Blotting Substrate (32,106, Thermo Scientific™) and imaged utilizing the Odyssey® Fc imaging system (Supplementary Figs. S1, S2, S3, S4).

### Gene expression analysis

The RNA isolation process was carried out using the Quick-RNA MicroPrep Kit from Zymo Research, adhering strictly to the manufacturer’s guidelines. RNA concentration in each sample was determined by measuring absorbance at 260 nm with a Nanodrop 2000 spectrophotometer (Thermo Fisher Scientific). To synthesize complementary DNA (cDNA), 800 ng of RNA was reverse transcribed using the M-MLV Reverse Transcriptase enzyme from Invitrogen. For the subsequent analysis of mRNA expression levels, quantitative reverse transcription PCR (qRT-PCR) was employed using the Light Cycler 480 SYBR Green I Master mix. Specific primer pairs for the target mRNAs, as listed in Table 1, were utilized in the qRT-PCR reactions. Each target gene was analyzed in triplicate to ensure accuracy and reproducibility of the results.

**Table 1:** Primer sequences of the m*Gapdh*, m*Gfrα1*, m*Ret*, m*Ncam*, and m*Met* genes used for quantitative real-time PCR

Gene	Primers (5′−3′)
*mGapdh*	Forward: GAGAAACCTGCCAAGTATG Reverse: GGAGTTGCTGTTGAAGTC
*mGfrα1*	Forward: ACTCCTGGATTTGCTGATGTCGG Reverse: CGCTGCGGCACTCATCCTT
*mRet*	Forward: CTGCCGCTGCTAGGAGAAGCCCCAC Reverse: CTTCACACTGATGTTGGGACAAAGGAA
*mNcam*	Forward: GCCGAGATGGTCATTCTGA Reverse: GATGTTGTCCAGGTGATGG
*mMet*	Forward: GTTCTGCTTGGCAACGAGAGCT Reverse: GGAGAATGCACTGTATTGCGTCG

### Statistical analysis

The experimental data were analyzed using descriptive statistics, including the calculation of means and standard error of the mean. Normality tests were conducted using the Shapiro–Wilk test. Non-parametric data (total tubule length, number of branches, qRT-PCR, and western blot) were analyzed using Kruskal–Wallis test followed by post-hoc Dunn’s multiple comparisons test. Data are presented as mean ± SEM. The statistical tests used for each dataset are specified in the figure legends. GraphPad Prism software version 9.1.2 was employed for all statistical analyses, and statistical significance was set at a *p*-value < 0.05.

## Results

### Induction of tubular structures by HGF

**Fig. 3:**
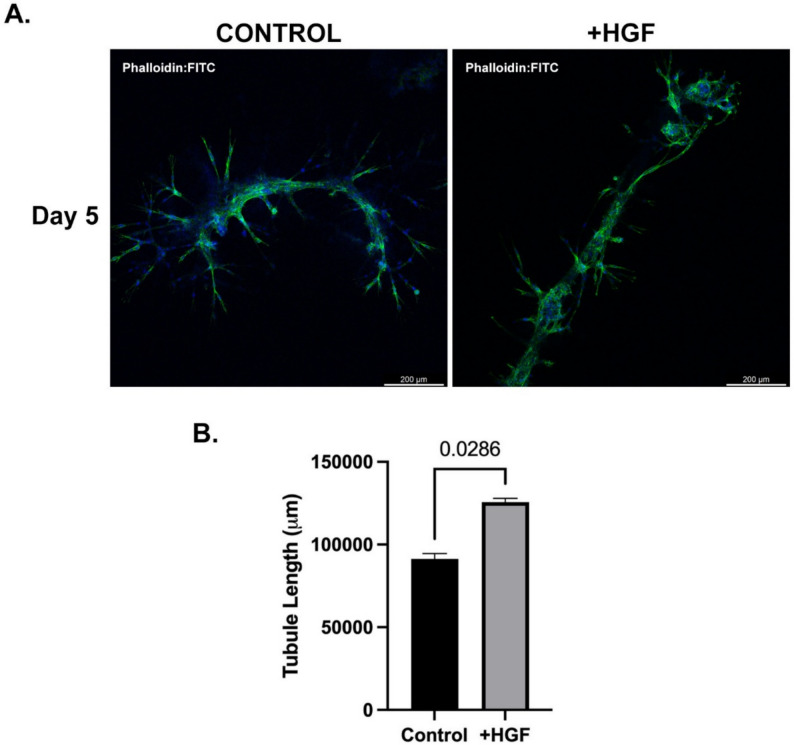
Staining of FITC-phalloidin to visualize actin staining in Sertoli cells after 5 days in 3D Matrigel-based culture. **A** Colocalization of phalloidin and DAPI staining are indicated in the merged images. Scale bar: 200 µm. **B** Quantitative analysis of tubule lengths between control and HGF (10 ng/mL) groups. Values are the median ± interquartile range (IQR)

In this study, we conducted an in vitro 3D Matrigel-based culture system to investigate the influence of HGF on the morphological changes of Sertoli cells through immunofluorescence staining of the cytoskeletal component. Sertoli cells were cultured in gel matrices in the presence of HGF, and their morphological alterations were examined (Fig. 3). Our observations revealed that Sertoli cells cultured in gels formed notably longer tubular-like structures in the presence of HGF compared to those cultured without HGF (*p* = 0.0286) (Fig. 3).

### Exogenous GDNF fortifies HGF-induced tubular-like structure formation

**Fig. 4:**
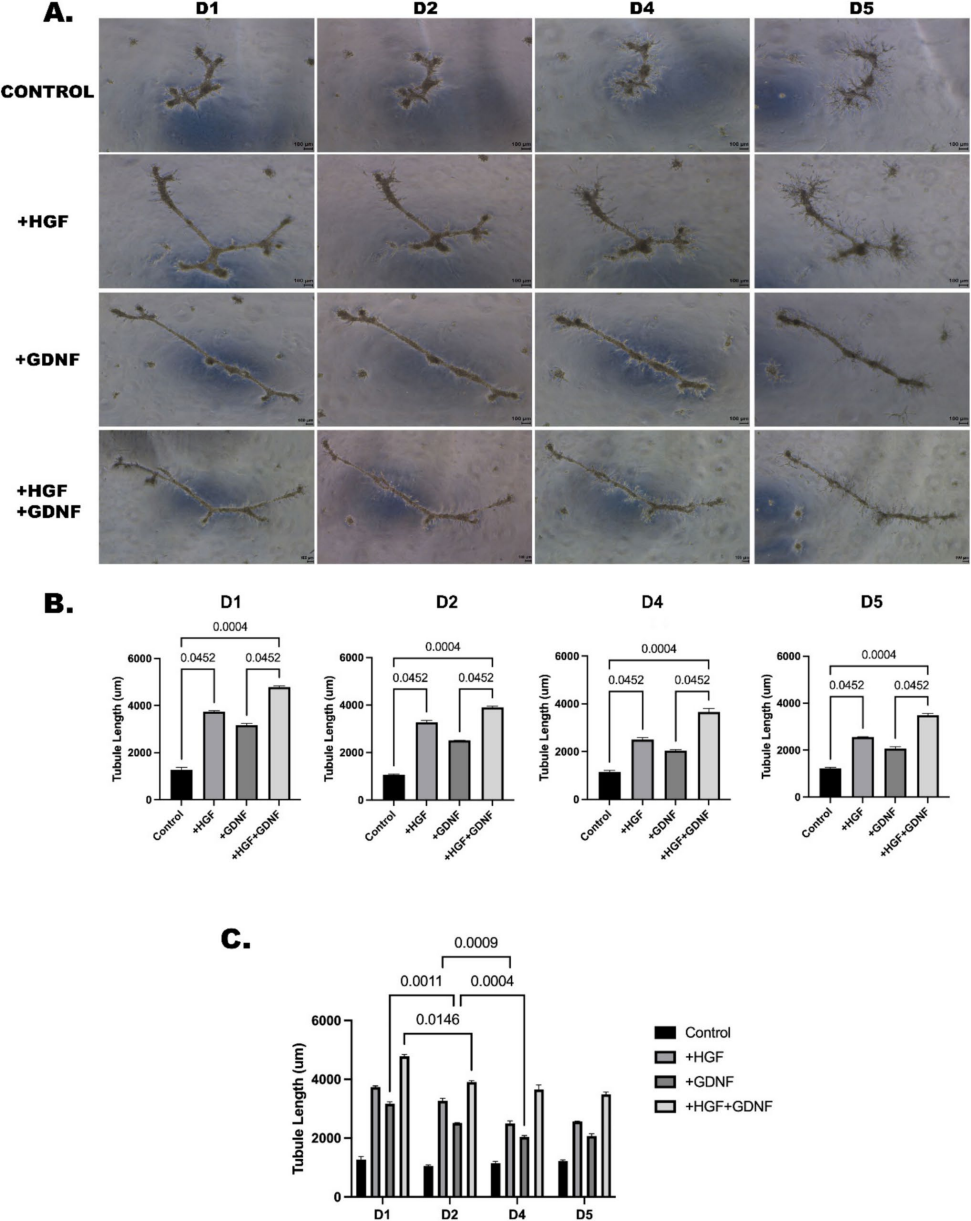
Enhancement of HGF-induced tubule formation of Sertoli cells by culture with exogenous GDNF. **A** Morphology of Sertoli cells cultured on Matrigel in the absence or presence of HGF (10 ng/mL) with GDNF (100 ng/mL) from day 1 (D1) to day 5 (D5). (Magnification, × 50). **B** Quantitative analysis of tubule lengths between all groups. Values are the median ± interquartile range (IQR) (*n* = 5). **C** Quantitative analysis of tubule lengths by day within each group. Values are the median ± IQR (*n* = 5)

**Fig. 5:**
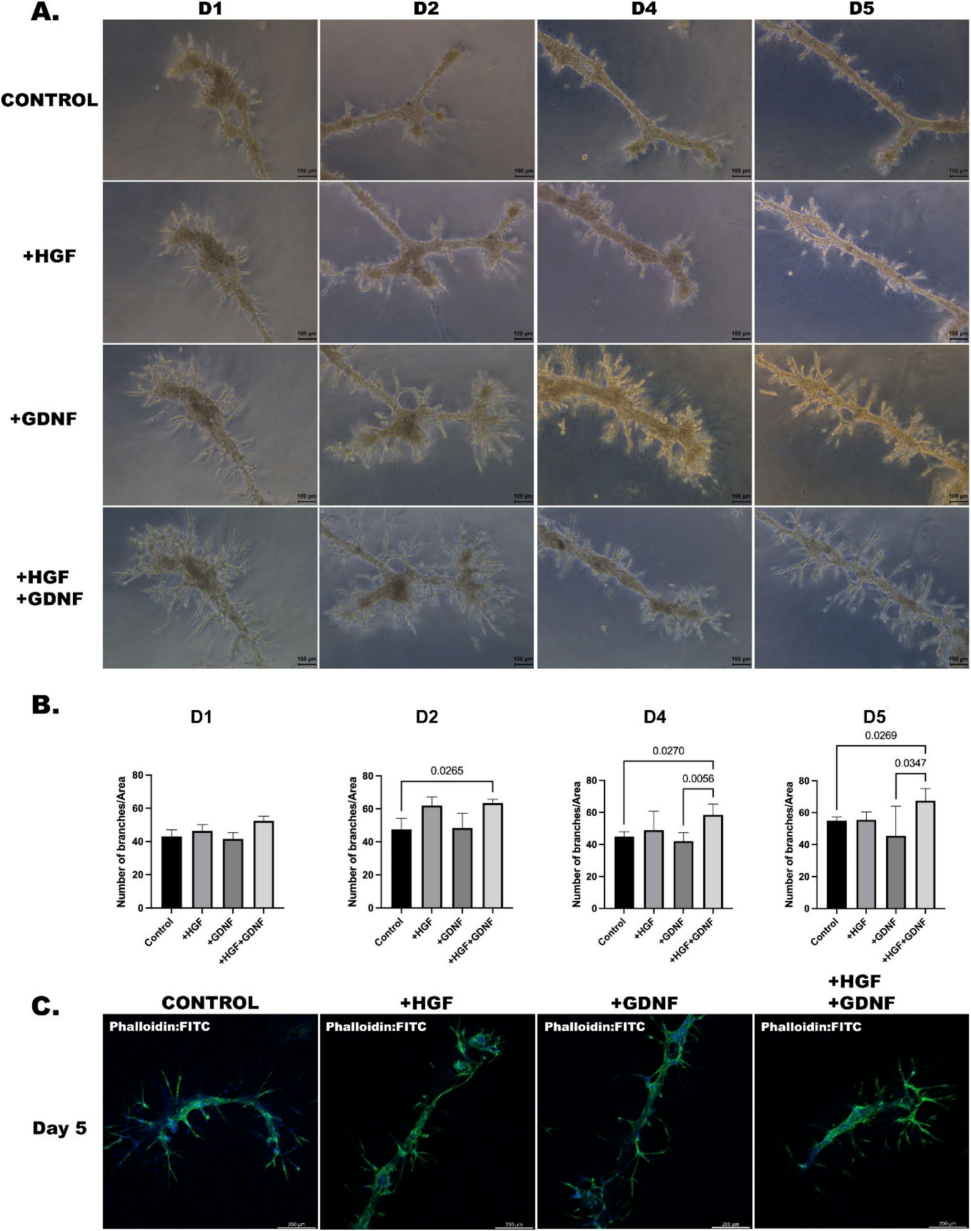
Morphology of HGF-induced tubule formation of Sertoli cells by culture with exogenous GDNF. **A** Branches of tubule-like structure of Sertoli cells cultured on Matrigel in the absence or presence of HGF (10 ng/mL) with GDNF (100 ng/mL) from day 1 (D1) to day 5 (D5). (Magnification, × 100). **B** Quantitative analysis of branch number per tubular structure. Values are the median ± IQR (*n* = 5). **C** Colocalization of phalloidin and DAPI staining are indicated in the merged images

To elucidate the impact of GDNF on HGF-induced tubular-like structure formation, we performed experiments involving the culture of Sertoli cells in gel matrices supplemented with HGF alone or in combination with GDNF. For 5 days, within the same time frame, our observations revealed a notable enhancement in length of HGF-induced tubular-like structure in the presence of GDNF compared to cultures lacking GDNF (Figs. 4 A and 5A). Throughout the experimental period, the inclusion of GDNF notably amplified the enhancement in tubule length induced by HGF when compared to the control group (*p* < 0.05) (Fig. 4B). Interestingly, no statistically significant difference was observed between the effects of HGF alone and the combined treatment with HGF and GDNF (Fig. 4B), suggesting a saturation effect or potential interaction between these growth factors in modulating Sertoli cell morphology and tubular-like structure formation. When the groups were compared within their respective time points, although there was a significant decrease in the group in which HGF and GDNF were added together until day 4, it was still significantly higher than the other groups (*p* < 0.05) (Fig. 4 C). When assessing the branching numbers, a substantial elevation was noted in the group administered with both HGF and GDNF starting from day 2, in comparison to the control group (*p* < 0.05) (Fig. 5B). Cytoskeleton staining was performed to visualize the three-dimensional structure of all groups and their branching patterns were observed (Fig. 5 C).

### Exogenous GDNF enhances the effect of forming tight junctions on HGF-induced tubular-like structures

**Fig. 6:**
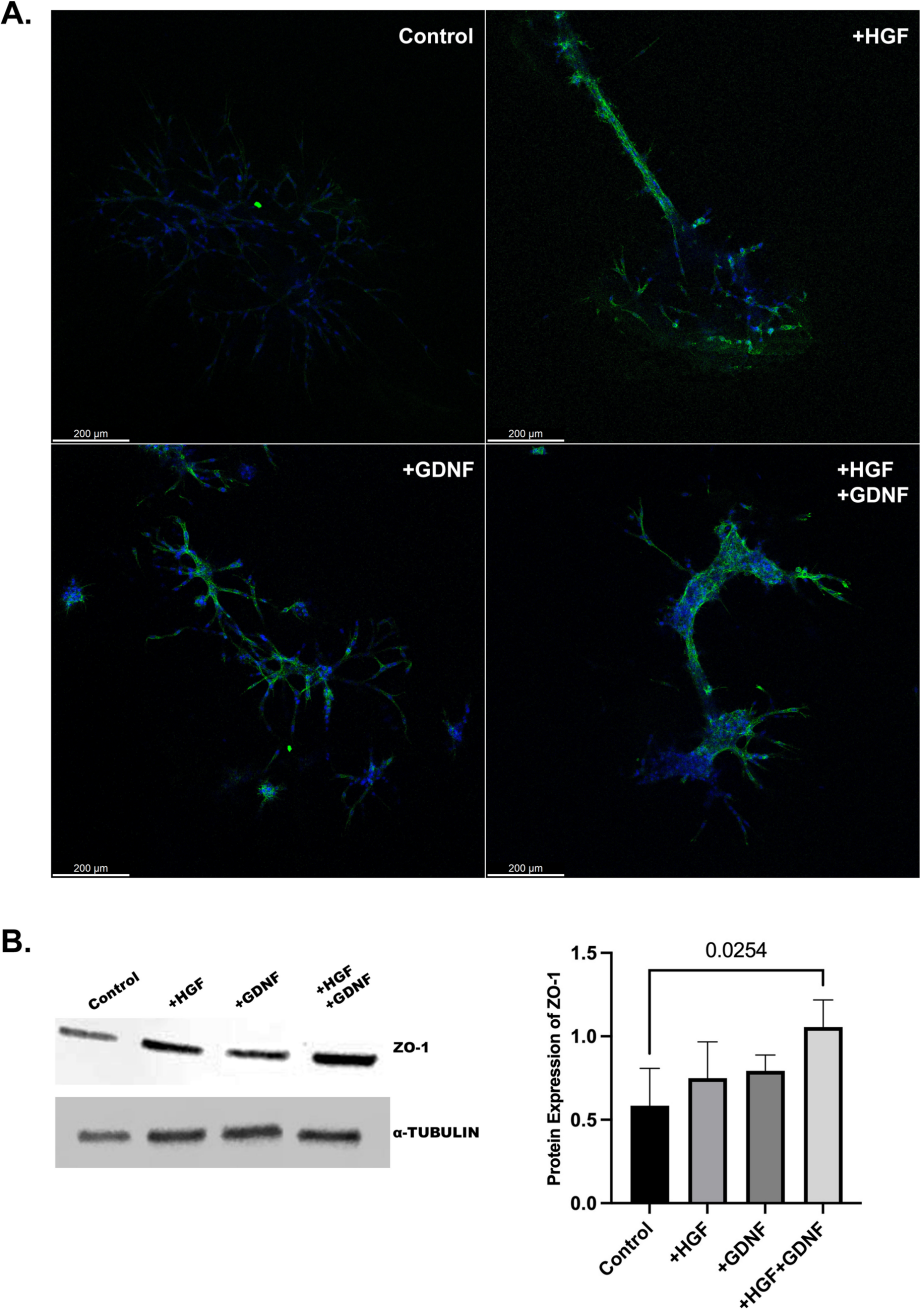
Expression of ZO-1 in tubule-like structures formed by the Sertoli cells in Matrigel after 5 days. **A** Colocalization of ZO-1 and DAPI staining is indicated in the merged images. **B** ZO-1 expression in Sertoli cells in 2D culture treated with HGF (10 ng/mL), GDNF (100 ng/mL), and HGF + GDNF. Values are the median ± IQR (*n* = 5)

In terms of the formation of the junctional complex of Sertoli cells, ZO-1 protein expression was evaluated by immunofluorescence and western blot method in all groups (Fig. 6). Immunofluorescence analysis of ZO-1 protein expression, a marker of tight junctions, revealed that both HGF and HGF in combination with GDNF led to more intense cytoplasmic ZO-1 expression compared to the control group (Fig. 6 A). Notably, tubule-like structures formed in the HGF + GDNF group exhibited prominent ZO-1 expression, indicative of robust tight junction establishment. Conversely, weak ZO-1 expression was observed in the GDNF group compared to the HGF and HGF + GDNF groups, suggesting a lesser effect of GDNF alone on tight junction formation. Western blot analysis further corroborated these findings, showing a significant increase in ZO-1 protein expression with the combined administration of GDNF and HGF, surpassing levels observed in the control group (*p* < 0.05) (Fig. 6B). These results underscore the synergistic role of GDNF in potentiating HGF-induced tight junction formation, highlighting its potential as a modulator of Sertoli cell intercellular dynamics and testicular function.

### Expression of Ret, NCAM, and GFRα1 in Sertoli cells

**Fig. 7:**
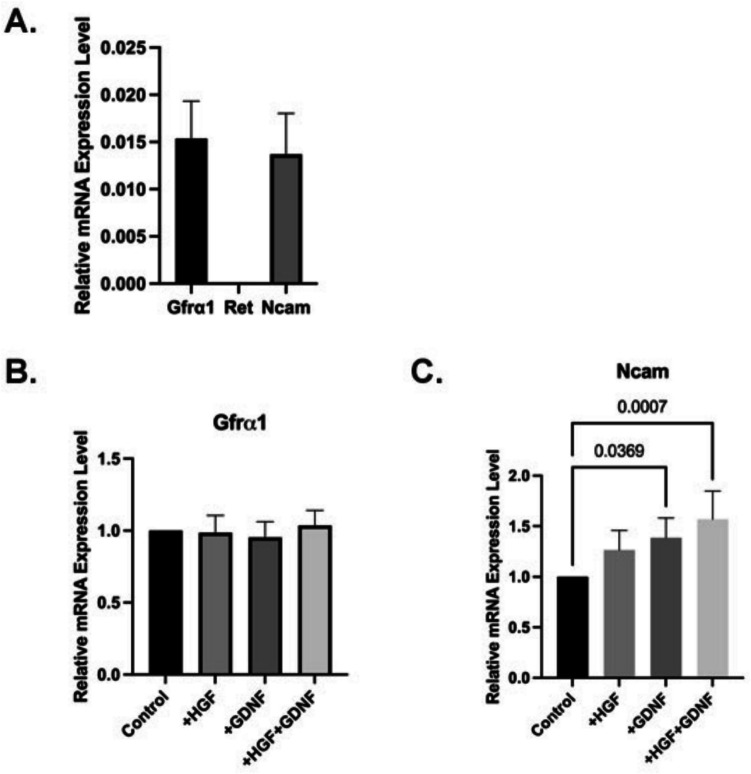
mRNA expression of Gfrα1, Ret, and NCAM in the Sertoli cells. **A** Relative quantification for mRNA expression level of Gfrα1, Ret, and NCAM in the Sertoli cells. **B-C** Gfrα1 and NCAM mRNA expression in Sertoli cells in 2D culture treated with HGF (10 ng/mL), GDNF (100 ng/mL), and HGF + GDNF. Values are the median ± IQR (*n* = 5)

We aimed to determine the presence of GDNF receptors, specifically Ret and GFRα1, present in Sertoli cells (Fig. 7 A). GDNF communicates via a multi-component receptor system that includes GFRα1 and one of the co-receptor subunits, either RET [19, 20] or NCAM [21, 22]. As the principal ligand-binding component, GFRα1 is necessary for the receptor complex to operate properly. The mRNA and protein levels of GFRα1 were verified by qRT-PCR investigations in Sertoli cells, but the mRNA expression of Ret was very low (Fig. 7 A). To confirm the specificity of the Ret primer and rule out any technical errors, mouse brain tissue was used as a positive control, demonstrating a high level of mRNA expression (Supplementary Fig. S5). Moreover, NCAM expression was also detected in Sertoli cells (Fig. 7 A). When Sertoli cells were cultured for 5 days and treated with HGF and GDNF, no significant difference in GFRα1 expression levels was observed among the different treatment groups (Fig. 7B). However, the presence of NCAM suggests it may mediate GDNF signal transduction in Sertoli cells. This finding highlights the potential role of NCAM in GDNF signaling, though further studies are needed to fully elucidate its involvement and the impact of other receptors and pathways.

### GDNF increases phosphorylation of HGF receptor c-Met in Sertoli cells

**Fig. 8:**
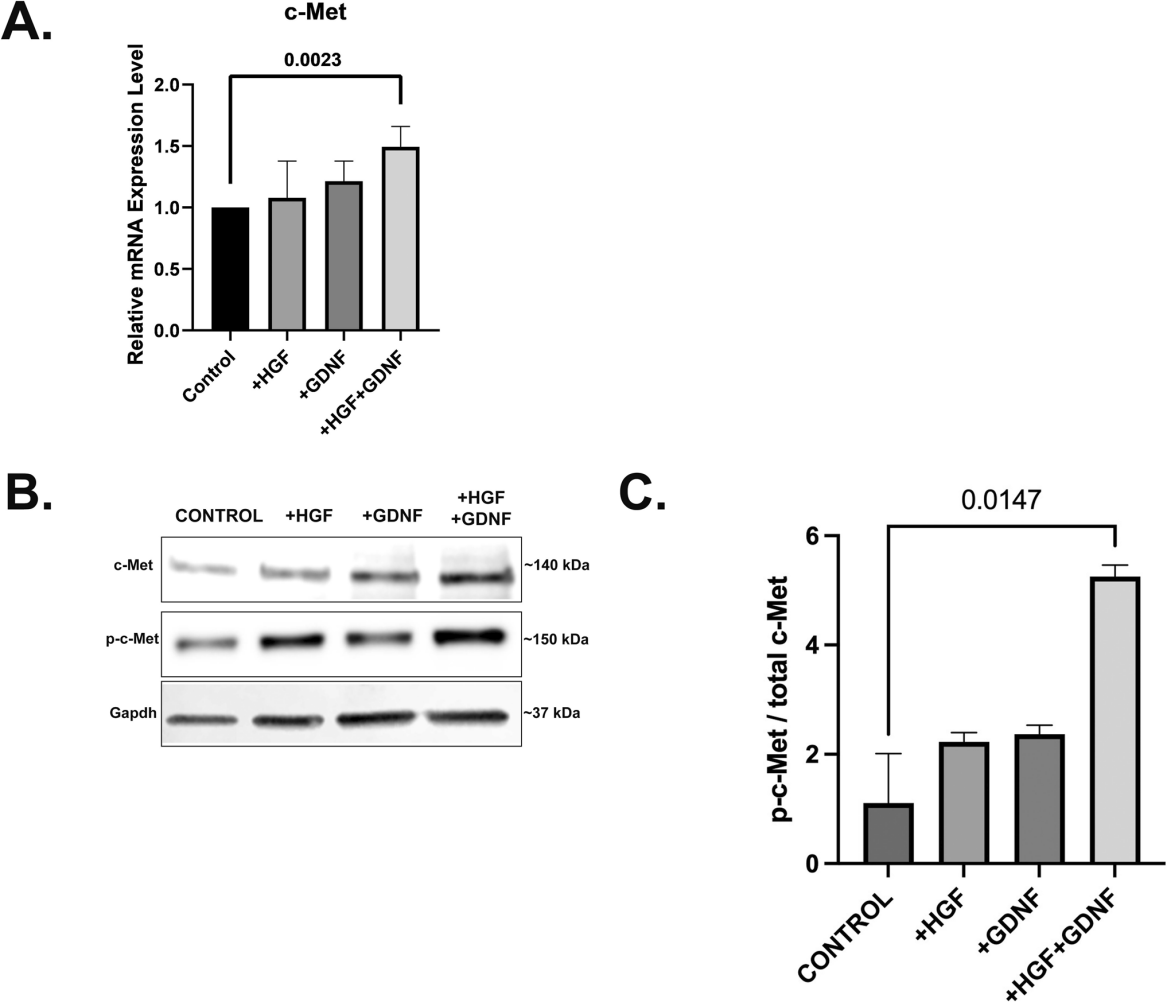
GDNF phosphorylates c-Met in Sertoli cells. **A** Relative quantification of Met mRNA expression in Sertoli cells in 2D culture treated with HGF (10 ng/mL), GDNF (100 ng/mL), and HGF + GDNF. **B** p–c-Met protein expression normalized with total c-Met protein expression in Sertoli cells in 2D culture treated with HGF (10 ng/mL), GDNF (100 ng/mL) and HGF + GDNF. **C** Quantitative analysis of c-Met and p–c-Met protein expressions. Values are presented as median ± IQR

To test the possibility that GDNF increases HGF-induced tubular-like structure formation by enhancing the HGF signaling pathway, we performed qPCR and western blotting to examine mRNA level of Met, total Met, and Met phosphorylation in Sertoli cells cultured in a monolayer (Fig. 8). Following 5 days of culture; we found significant increase in Met mRNA expression when HGF and GDNF were administered together. On the other hand, we found a significant increase in phospho-Met levels in Sertoli cells treated with HGF alone, indicating activation of the HGF signaling pathway (*p* < 0.05) (Fig. 8B). Interestingly, stimulation with GDNF alone did not lead to a notable augmentation in Met phosphorylation by the end of the 5-day period. Furthermore, quantitative analysis revealed that co-administration of HGF and GDNF significantly elevated phospho-Met levels compared to the control group (*p* < 0.05) (Fig. 8B). These results suggest that while GDNF alone may not directly induce Met phosphorylation, its co-administration with HGF enhances Met activation, indicating a potential synergistic effect between these growth factors in modulating Sertoli cell signaling pathways.

## Discussion

**Fig. 9:**
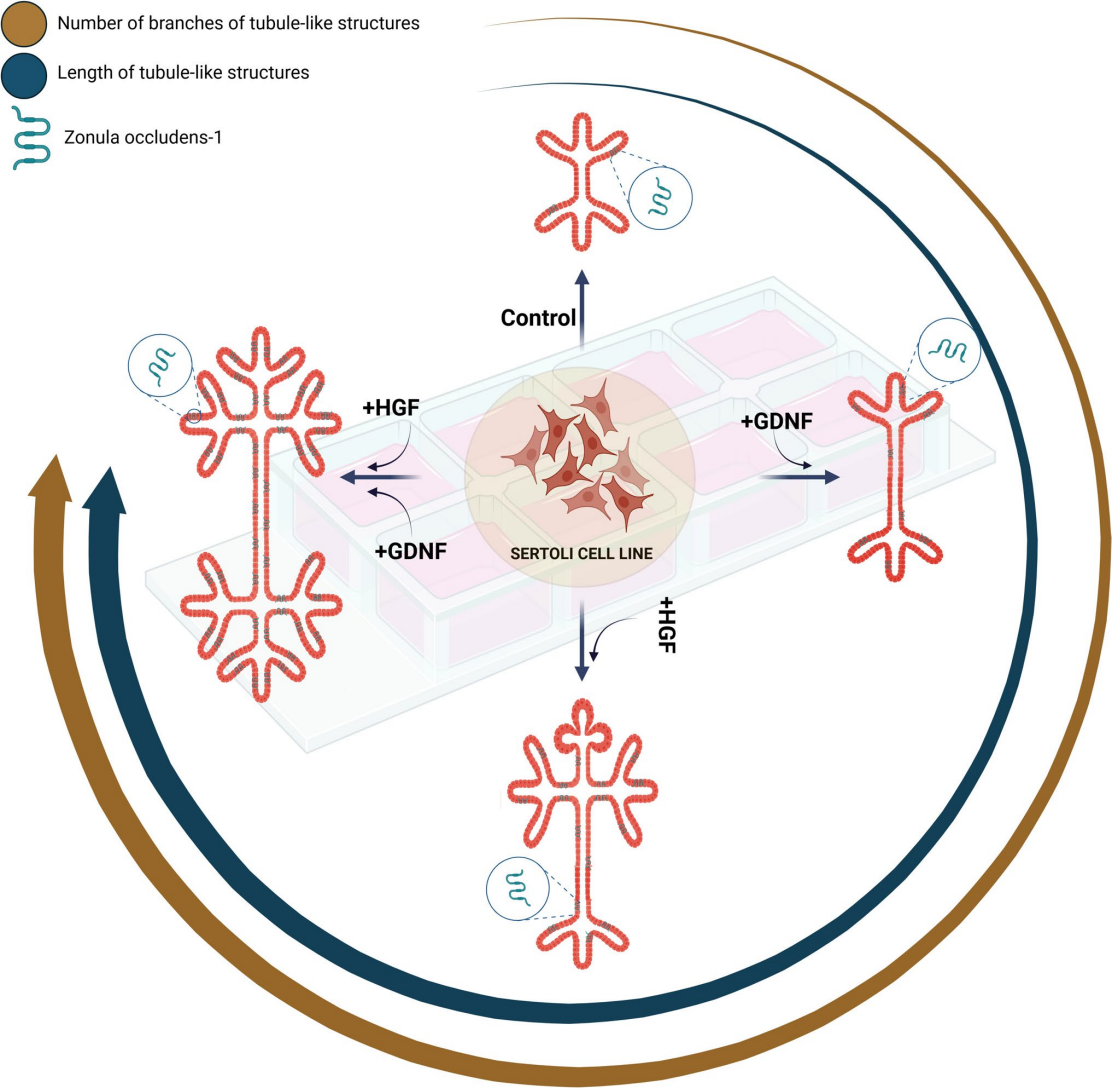
Summary diagram. Schematic representation of the interplay between HGF and GDNF in modulating Sertoli cell morphology and intercellular dynamics, emphasizing their collective impact on tubular structure formation and tight junction establishment. The illustration was generated using the BioRender software

The findings of the present study shed light on the role of HGF and GDNF in orchestrating morphological changes and the formation of tubular-like structures in Sertoli cells within an in vitro 3D Matrigel-based culture system (Fig. 9). By examining Sertoli cell behavior in response to HGF, we observed a substantial augmentation in the length and formation of tubular-like structures, indicative of HGF's inducible effect on cellular morphology. Furthermore, the addition of GDNF alongside HGF enhanced tubular structure more than HGF alone. However, despite the observed morphological changes, the inclusion of GDNF did not result in a significant difference compared to HGF alone, suggesting that GDNF by itself does not have an effect. Moving beyond morphological alterations, we delved into the intricate realm of intercellular junction formation, particularly focusing on the expression of ZO-1 protein, a hallmark of tight junctions. Notably, both HGF and the combination of HGF with GDNF fostered robust ZO-1 expression, indicative of tight junction establishment between Sertoli cells. In contrast, GDNF alone exhibited a weaker increase in ZO-1 expression compared to the combined treatment. Furthermore, we explored the underlying molecular mechanisms by examining Met phosphorylation levels in response to HGF and GDNF stimulation. While GDNF alone did not induce significant Met phosphorylation, its co-administration with HGF resulted in a significant elevation in phospho-Met levels compared to the control group, suggesting a potential synergistic effect between these growth factors in modulating Sertoli cell signaling pathways. These findings collectively underscore the intricate interplay between HGF and GDNF in orchestrating morphological changes and intercellular dynamics within the Sertoli cell microenvironment.

The rationale behind employing HGF to induce Sertoli cells to form tubules is grounded in its well-established role in testicular development and spermatogenesis [23–26]. HGF, along with its receptor c-Met, plays critical roles in various aspects of testicular morphogenesis and function, including the transformation of testicular cords into tubules and the regulation of tight junction formation between Sertoli cells [27–29]. Additionally, HGF’s ability to modulate cellular polarity and promote consistent cellular organization makes it an ideal candidate for inducing Sertoli cell tubulogenesis in vitro [14]. Our study utilized an in vitro 3D Matrigel-based culture system to investigate the influence of HGF on Sertoli cell morphology. We observed that HGF significantly increased the formation of tubular-like structures compared to control conditions, highlighting its importance in modulating Sertoli cell morphology and tubule formation.

GDNF expression is found to be highly expressed in the testis during critical developmental stages, suggesting its importance in coordinating events such as Sertoli cell proliferation and cord formation [30, 31]. Studies have shown that GDNF has a stimulatory effect on the proliferation and cord formation of embryonic Sertoli cells, indicating its involvement in testicular morphogenesis [30, 31]. Additionally, it is hypothesized that GDNF, along with other growth factors, may regulate signaling cascades involved in cell proliferation, differentiation, and cell–cell interactions during testicular development [15, 32, 33]. However, despite the evidence suggesting the significance of GDNF in testicular morphogenesis, there remains a lack of comprehensive information regarding its specific effects and mechanisms of action. Therefore, further investigation is warranted to elucidate the role of GDNF in testicular development and function, particularly in the context of Sertoli cell biology and tubule formation. We also investigated the synergistic impact of exogenous GDNF and HGF in Matrigel-based 3D culture.

In our study, we employed length and branching number assessments to evaluate the impact of GDNF on HGF-induced tubular-like structure formation in Sertoli cells cultured in gel matrices. Using of length and branching number assessments aligns with established methodologies in tube formation assays, where quantification involves measuring the total tube length and the number of branch points [34, 35]. Specifically, by quantifying the length and branching number of tubular-like structures in our experimental conditions, we could objectively assess the extent of tubule formation and branching in response to HGF and GDNF treatments. Our findings demonstrated a significant increase in the length and branching number of tubular-like structures when GDNF was co-administered with HGF compared to cultures treated solely with HGF. This quantitative analysis provided valuable insights into the synergistic effects of HGF and GDNF on Sertoli cell morphology and tubular-like structure formation, suggesting potential interactions between these growth factors in modulating tubule formation. Moreover, the lack of significant difference between the effects of HGF alone and the combined treatment with HGF and GDNF implies a saturation effect or potential complex interplay between these factors in regulating Sertoli cell morphology and tubule formation, highlighting the importance of quantitative assessments in elucidating cellular responses to growth factor treatments.

Previous studies [36, 37] have shown that branching and anastomosis are inherent features of seminiferous tubule development in embryonic and postnatal testes. Similarly, branching points have been described in detailed 3D reconstructions of adult mouse seminiferous tubules [38], suggesting that such structures are not uncommon. In our study, the branching observed after HGF + GDNF treatment may reflect a temporary alteration in tubule architecture influenced by these growth factors. HGF and GDNF are both known to affect cellular organization, and their combined effect may induce localized remodeling, particularly in Sertoli cells, which play a key role in tubule structure. While the branches observed in our study were not sustained long-term, their transient nature might indicate an adaptation during the experimental conditions rather than a disruption of normal development.

There is the pivotal role of tight junctions, which include multiple integral membrane proteins in the formation of tubule lumens by Sertoli cells [39]. ZO-1 serves as a critical molecular marker for monitoring inter-Sertoli junction formation, making it a suitable target for assessing the integrity of tight junctions [40, 41]. In our study, we evaluated ZO-1 protein expression through immunofluorescence and western blot methods across experimental groups. Our results revealed that both HGF and HGF combined with GDNF treatment led to intensified cytoplasmic ZO-1 expression compared to the control group. Notably, tubule-like structures formed in the HGF + GDNF group exhibited prominent ZO-1 expression, indicative of robust tight junction establishment. Conversely, weaker ZO-1 expression was observed in the GDNF group compared to the HGF and HGF + GDNF groups, suggesting a lesser effect of GDNF alone on tight junction formation. Western blot analysis further supported these findings, demonstrating a significant increase in ZO-1 protein expression with the combined administration of GDNF and HGF, surpassing levels observed in the control group. These results underscore the synergistic role of GDNF in potentiating HGF-induced early-stage junction formation, highlighting its possible potential as a modulator of Sertoli cell intercellular dynamics and testicular function. As a limitation, we acknowledge that while the increase in ZO-1 expression may suggest early-stage junction formation, further validation using additional techniques, such as electron microscopy or functional assays, is required to confirm the functional relevance of these structures.

Our findings indicate that Sertoli cells in the 3D culture system organize into tubule-like structures through cell–cell interactions rather than simple aggregation. However, these structures do not exhibit fully developed lumens, and thus, we refer to them as tubule-like structures. This organization was confirmed through phase-contrast microscopy and immunostaining, which demonstrated the polarization of Sertoli cells. Notably, a significant difference in tubule length was observed as early as day 1, suggesting that the experimental conditions facilitated a more rapid structural organization of Sertoli cells. We hypothesize that this early increase in tubule length is primarily due to alterations in cell–cell interactions and morphological changes rather than increased cell migration. These findings highlight the dynamic nature of Sertoli cell organization in a 3D environment and underscore the importance of microenvironmental factors in testicular tissue.

The literature highlights the intricate interplay between GDNF/GFRα1/MET signaling pathways in various cellular contexts, shedding light on their diverse roles in cellular differentiation and signaling regulation [42, 43]. Additionally, the synergistic activation of mitogenic signaling cascades mediated by protein kinases ERK1/2 upon co-administration of HGF and GDNF suggests a potential mechanism for enhancing cellular proliferation and neurite outgrowth [43]. Additionally, it has been demonstrated that GDNF activates the HGF receptor c-Met in MDCK cells’ culture environment, inducing ureteric branching without utilizing the RET signal [16]. Moreover, GDNF derived from HUVECs has been shown to enhance the in vitro renal tubule formation capacity of HGF [17]. Our study contributes to this body of knowledge by evaluating the effects of HGF and GDNF on Sertoli cell receptors at the protein and mRNA level in a 2D Sertoli cell culture model. In Sertoli cells, when examining the expression of the GDNF receptor Gfra1, Ret, and the co-receptor known as NCAM, it was shown that there is no expression of Ret. This suggests that Sertoli cells interact through different pathways instead of using Ret for GDNF. Interestingly, while GDNF alone did not directly induce Met phosphorylation, its co-administration with HGF led to a significant increase in phospho-Met levels, indicating a potential synergistic effect between these growth factors in modulating Sertoli cell signaling pathways. These findings provide valuable insights into the complex molecular mechanisms underlying tubular structure formation by Sertoli cells and highlight the potential therapeutic implications of targeting the GDNF/HGF signaling axis in reproductive biology and male fertility.

In conclusion, we investigated the effects of growth factors HGF and GDNF on Sertoli cell organization and testicular tubule formation, offering valuable insights into their role in male reproductive health. Our findings demonstrate that HGF significantly enhances tubular structure formation by Sertoli cells, and when combined with GDNF, this effect is further potentiated. We also observed robust ZO-1 expression, indicating early-stage junction formation, alongside evidence of potential synergistic effects between HGF and GDNF in modulating Sertoli cell signaling. These results provide a deeper understanding of Sertoli cell morphology and function, with potential implications for developing therapeutic strategies in male infertility, tissue regeneration, and testicular repair. By advancing our knowledge of Sertoli cell dynamics, this study paves the way for novel regenerative approaches aimed at restoring spermatogenesis and improving male fertility, particularly in conditions like azoospermia and testicular atrophy.

## Supplementary Information

Below is the link to the electronic supplementary material.

Supplementary file1 (JPG 44.4 KB)

Supplementary file2 (JPG 27.8 KB)

Supplementary file3 (JPG 201 KB)

Supplementary file4 (JPG 44.4 KB)

Supplementary file5 (JPG 65.5 KB)

## Data Availability

The data supporting the findings of this study are available from the corresponding author upon reasonable request.
