# Supplementary figures and images for: GDNF enhances HGF-induced tubulogenesis and organization of Sertoli cell
Source: J Assist Reprod Genet. 2025 May 22;42(6):2083–98. doi: 10.1007/s10815-025-03493-7 (PMC12229439; doi:10.1007/s10815-025-03493-7)

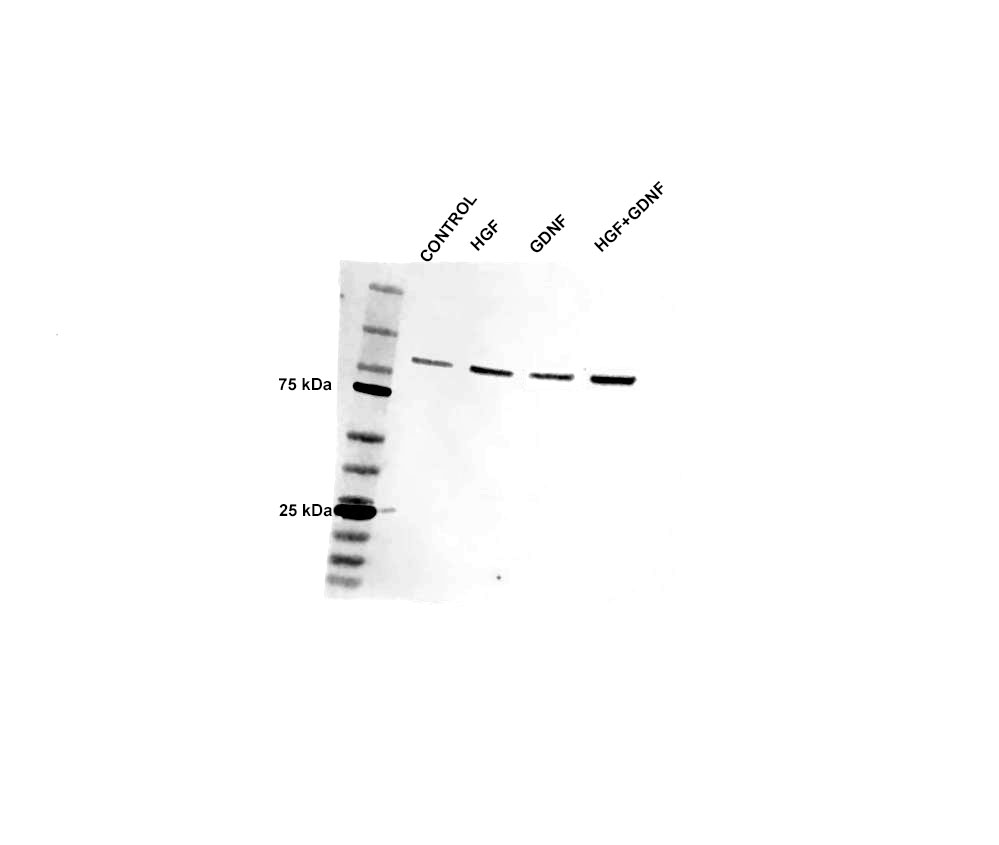

Supplement: Supplementary file 1 — Supplementary file1 (JPG 44.4 KB) [file 10815_2025_3493_MOESM1_ESM.jpg]

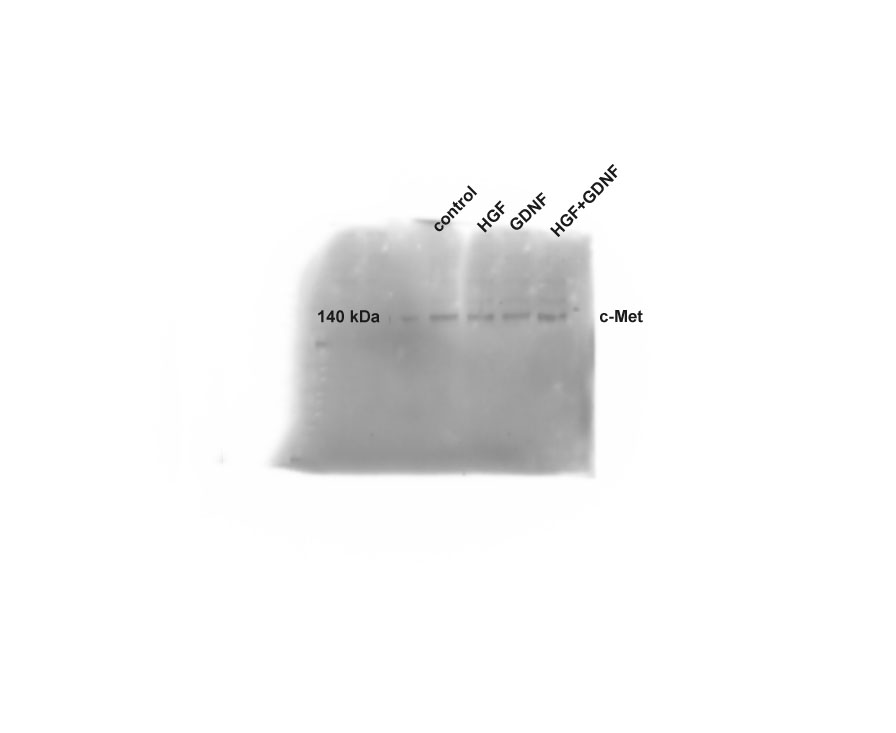

Supplement: Supplementary file 2 — Supplementary file2 (JPG 27.8 KB) [file 10815_2025_3493_MOESM2_ESM.jpg]

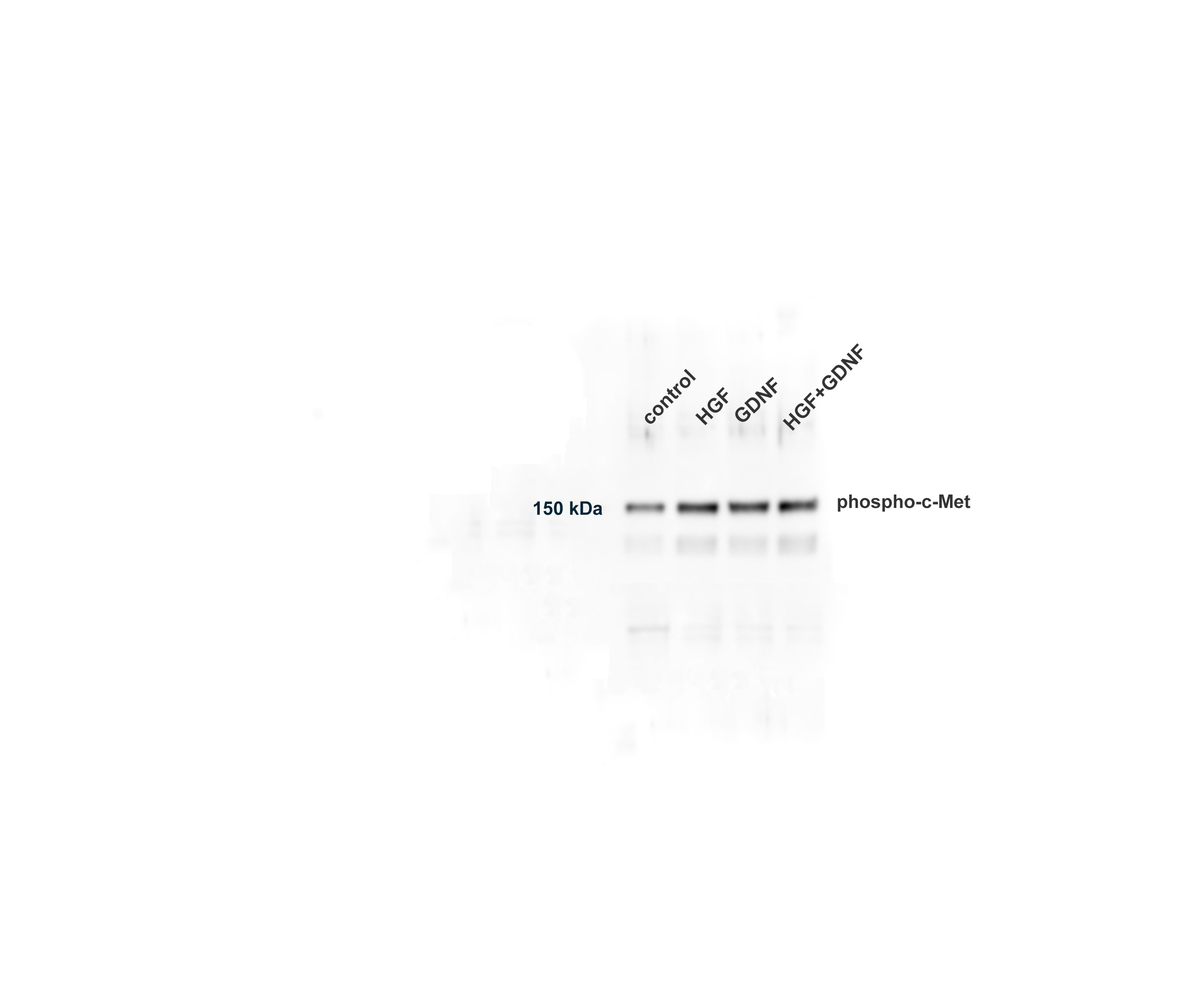

Supplement: Supplementary file 3 — Supplementary file3 (JPG 201 KB) [file 10815_2025_3493_MOESM3_ESM.jpg]

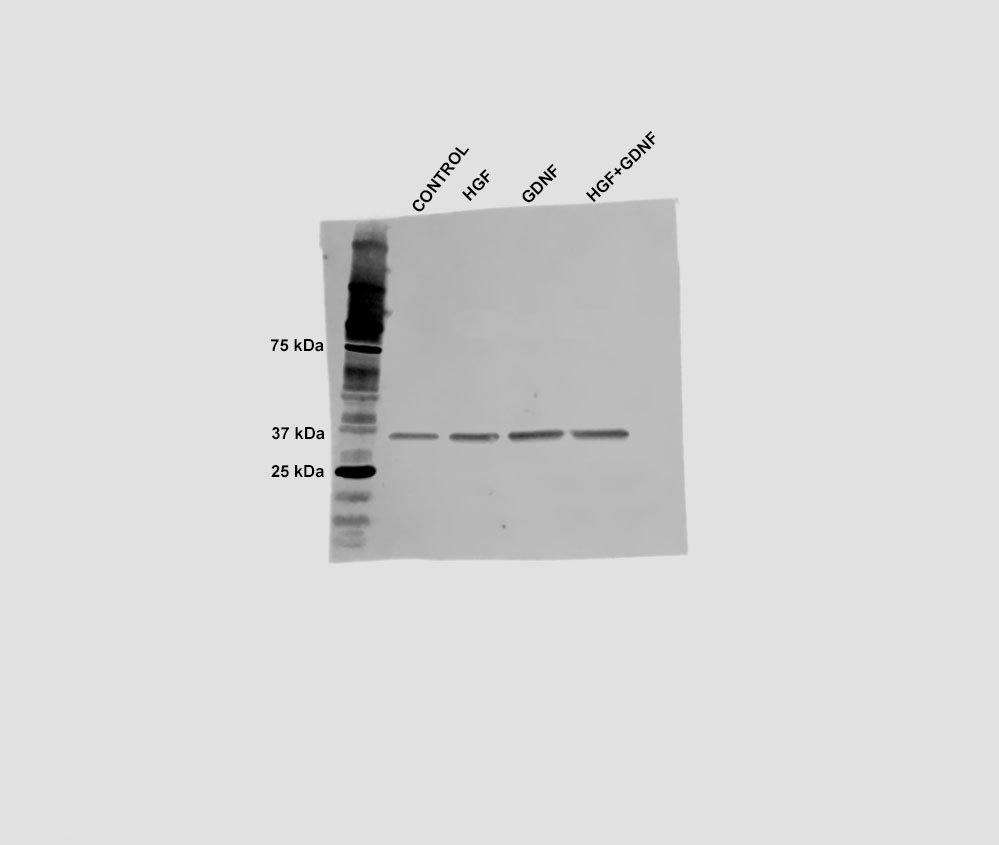

Supplement: Supplementary file 4 — Supplementary file4 (JPG 44.4 KB) [file 10815_2025_3493_MOESM4_ESM.jpg]

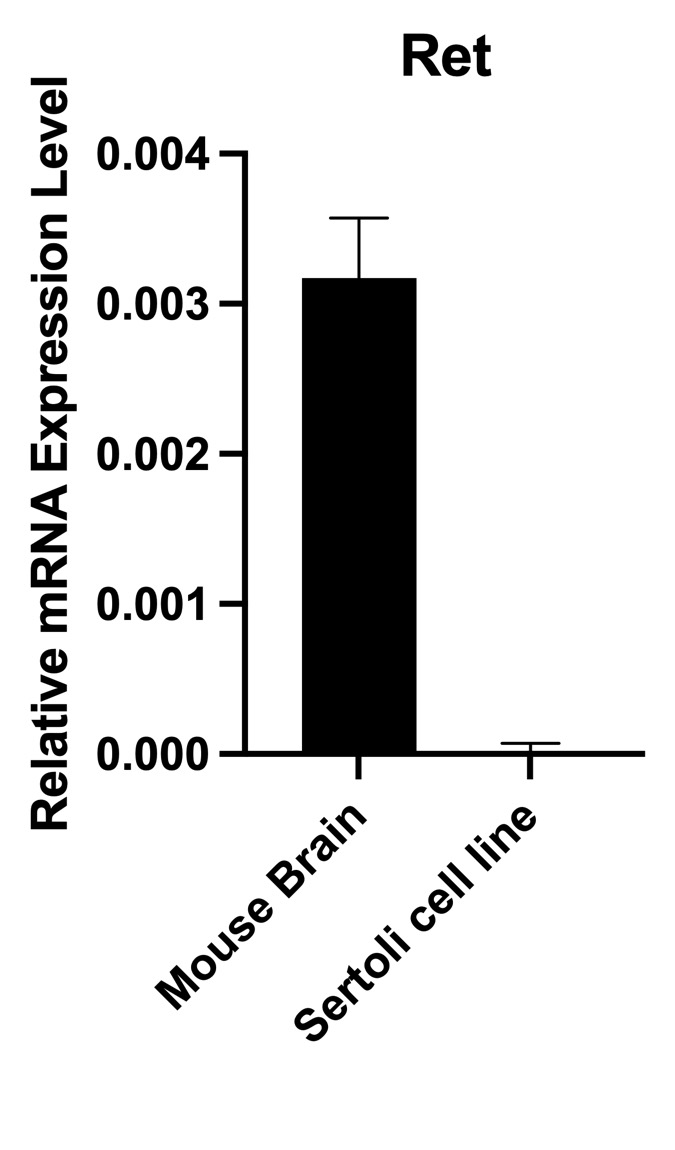

Supplement: Supplementary file 5 — Supplementary file5 (JPG 65.5 KB) [file 10815_2025_3493_MOESM5_ESM.jpg]
